# Automatic analysis of the 3-D microstructure of fruit parenchyma tissue using X-ray micro-CT explains differences in aeration

**DOI:** 10.1186/s12870-015-0650-y

**Published:** 2015-10-30

**Authors:** Els Herremans, Pieter Verboven, Bert E. Verlinden, Dennis Cantre, Metadel Abera, Martine Wevers, Bart M. Nicolaï

**Affiliations:** BIOSYST-MeBioS, KU Leuven, Willem de Croylaan 42, 3001 Leuven, Belgium; Flanders Centre of Postharvest Technology, Willem de Croylaan 42, 3001 Leuven, Belgium; MTM, KU Leuven, Kasteelpark Arenberg 44, 3001 Leuven, Belgium

**Keywords:** Image analysis, Apple, Pear, Diffusion, Oxygen, Gas space, Mathematical model, Tomography

## Abstract

**Background:**

3D high-resolution X-ray imaging methods have emerged over the last years for visualising the anatomy of tissue samples without substantial sample preparation. Quantitative analysis of cells and intercellular spaces in these images has, however, been difficult and was largely based on manual image processing. We present here an automated procedure for processing high-resolution X-ray images of parenchyma tissues of apple (*Malus* × *domestica* Borkh.) and pear (*Pyrus communis* L.) as a rapid objective method for characterizing 3D plant tissue anatomy at the level of single cells and intercellular spaces.

**Results:**

We isolated neighboring cells in 3D images of apple and pear cortex tissues, and constructed a virtual sieve to discard incorrectly segmented cell particles or unseparated clumps of cells. Void networks were stripped down until their essential connectivity features remained. Statistical analysis of structural parameters showed significant differences between genotypes in the void and cell networks that relate to differences in aeration properties of the tissues.

**Conclusions:**

A new model for effective oxygen diffusivity of parenchyma tissue is proposed that not only accounts for the tortuosity of interconnected voids, but also for significant diffusion across cells where the void network is not connected. This will significantly aid interpretation and analysis of future tissue aeration studies. The automated image analysis methodology will also support pheno- and genotyping studies where the 3D tissue anatomy plays a role.

**Electronic supplementary material:**

The online version of this article (doi:10.1186/s12870-015-0650-y) contains supplementary material, which is available to authorized users.

## Background

Bulky plant organs such as pome fruit mainly consist of parenchyma tissue, that is important for metabolic processes such as respiration. Aeration [1] of the parenchyma is essential to maintain the cellular metabolism of the fruit [2, 3]. Values of the effective oxygen diffusivity of fruit tissues have been shown to be very low, limiting respiration that could lead to ATP deficiency in low oxygen conditions [4]. Furthermore, significant differences in oxygen diffusivity of fruit parenchyma have been observed between fruit genotypes [5, 6]. Knowledge of aeration properties thus helps understanding of fruit physiology.

Fruit parenchyma can be regarded as a porous medium with air spaces distributed in between the cells. Porous media theory states that the effective diffusivity of the tissue can be calculated from molecular diffusivity multiplied with porosity and divided by a tortuosity factor [7–10]. Tortuosity accounts for the fact that gasses follow the meandering network of air spaces in the tissue rather than a straight path. While tissue porosity can be easily measured, tortuosity depends on the structure of the pore network and is more difficult to quantify. Usually tortuosity is determined inversely from the measured diffusivity as was already applied to gas diffusion in fruit [10]. Attempts using different predictive methods for tortuosity from porosity [7–9] lead to effective diffusivity values that are orders of magnitude larger than those measured on fresh tissue samples. It is hypothesized that the structure of plant tissue differs significantly from traditional porous media leading to lower effective diffusivity values than theoretically expected. Indeed, tissues are not necessarily homogeneous, isotropic and random, and diffusion occurs also through the cells [3]. It can be expected that tortuosity then is not a function of porosity but that other structure characteristics play a role. It is unclear which tissue characteristics are determinant. There is thus a need to quantify structural characteristics of cells and voids in the tissues that affect the effective oxygen diffusivity of parenchyma tissue.

Plant tissue microstructure has conventionally been visualized using light microscopy [11, 12], possibly with chemical staining [13, 14] or labelling [15], cryo-SEM [16] or ESEM [17]. These techniques require invasive sample preparation and/or preprocessing steps that are time consuming and may introduce artefacts in the images. Also, as these techniques are essentially based on 2-D slices of images, sample coverage is restricted by the number of sections that are taken. Further, the sectioning angle may lead to wrong estimates of cell diameters or other microstructural features such as the connectivity of the intercellular air space [3, 6]. Confocal laser scanning microscopy allows the production of 3D images with resolutions even beyond the refraction limit. However, even in multiphoton systems their penetration depth is limited to below 1 mm, and high resolution images require fluorescent probes to be inserted in the cells [18].

X-ray micro-computed tomography (X-ray micro-CT) has emerged as an attractive 3D imaging tool for plant anatomy research with important advances over other methods [6, 19–21]. X-ray micro-CT can visualize cells and intercellular spaces of tissue samples that need no other pretreatment than cutting from the fruit and securing in the sample holder of the X-ray CT device, rendering an image at a resolution in the order of 1 micrometer in a few tens of minutes. This results in sufficiently high quality images of the intercellular space in between cells, but cell wall boundaries between cells are not resolved. When phase contrast imaging is applied [22, 23], walls may become slightly visible but automatic isolation of individual cells remains impossible and thus have to be segmented by tedious manual operations. As a result, identification and quantification of individual cells is not straightforward, unless contrast is enhanced with staining methods [24], which increases sample preparation times considerably. Processing tomographic images of fresh samples in a standardized manner can be a great challenge [23, 25, 26], but is required for quantitative analysis of size, shape and connectivity of cells and voids in between cells. An automated cell analysis algorithm for X-ray tomographic images is not yet available and will enhance the more wide use of the method to assist understanding of aeration of plants.

As a result, although large datasets of microtomography images could be obtained, progress needs to be made with image processing, which is often a major hurdle for any visualization method to be successful. We presumed that X-ray micro-CT images of plant parenchyma tissue outline cell borders touching intercellular spaces sufficiently such that the outline could be completed using this information with advanced image processing techniques.

The aim of the current study was to (1) develop a method for automatically identifying and characterizing individual cells and voids in 3-D images of parenchyma tissue of apple and pear fruit obtained by desktop and synchrotron X-ray CT; and (2) to use the method to improve understanding of gas exchange properties of pome fruit parenchyma. The developed methodology is equally applicable to analysis of parenchyma properties in other plant organs. We chose four distinct pome fruit genotypes of economic relevance that have different responses to hypoxic conditions [4]. ‘Conference’ pear and ‘Braeburn’ apple are particularly sensitive to develop storage disorders in low oxygen conditions; ‘Jonagold’ is much less sensitive and ‘Kanzi’ is a relatively new cultivar that has been little studied. Here we verify whether these differences are related to changes in aeration caused by parenchyma tissue structure differences.

## Results

### Characteristics of parenchyma cells in pome fruit cortex

**Fig. 1:**
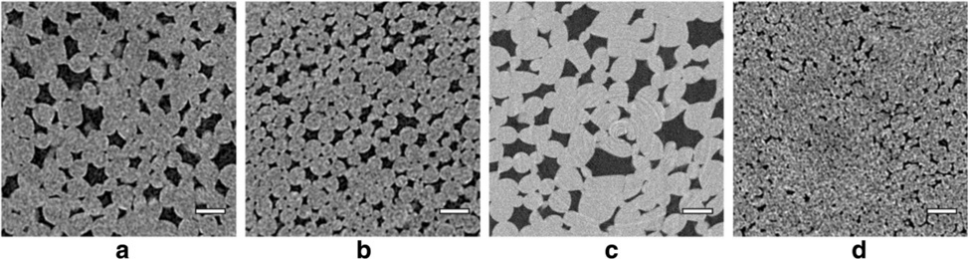
Original X-ray micro CT virtual cross-sectional images of ‘Braeburn’ (**a**), ‘Kanzi’ (**b**), ‘Jonagold’ (**c**) and ‘Conference’ (**d**) microstructure, obtained by means of a desktop CT system (**a**, **b**, **d**) or in a large scale synchrotron radiation CT facility (**c**). Cells and intercellular space are visible in all the images. Scale bar indicates 250 μm

**Fig. 2:**
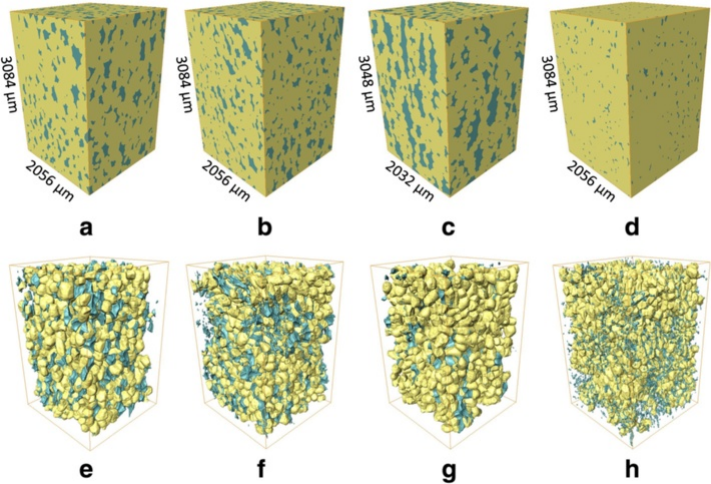
Bulk microstructure model of ‘Braeburn’ (**a**), ‘Kanzi’ (**b**), ‘Jonagold’ (**c**) apple and ‘Conference’ (**d**) pear tissue, and 3D model of the same samples resp. **e f g h** after the automatic isolation protocol for cells (yellow) and voids (blue). The dimensions of the analyzed datastacks are presented in μm, and are the same for the bulk microstructure models as for the isolated void and cell models

Images of fruit cortex parenchyma obtained at 5 μm pixel resolution can resolve characteristic features of the cell architecture and air spaces of apple and pear tissue (Fig. 1). By applying the cell isolation protocol we were able to quantify a large amount of individual cells (500-1500) from each 3D image. This numerical sieve allowed quantitative analysis and statistical testing of differences in cell size and shape, which was previously not possible [6]. ‘Jonagold’ cells are on average the largest with an equivalent spherical diameter equal to 210 μm, whereas ‘Conference’ cells were the smallest (159 μm) (Table 1). Cell sizes of ‘Braeburn’ (197 μm) and ‘Jonagold’ do not differ significantly, while ‘Kanzi’ apple cells (172 μm) are similar in size to those of ‘Conference’ pear. We were able to fit normal distributions to the measured cell volumes using the algorithm, as plotted in Fig. 2 (*p*-values of normal > 0.05). The number density of cortex cells for the different genotypes (Table 1) have the same statistical differences: ‘Kanzi’ and ‘Conference’ have a significantly larger number of cells than ‘Jonagold’ and ‘Braeburn’.

**Table 1:** Structural parameters of cortex fruit tissue of apple and pear genotypes (mean ± standard deviation). A large amount of cells and voids were isolated and measured in 4 samples for each genotype. Different letters indicate differences at 5 % significance level

Parameter	‘Braeburn’	‘Kanzi’	‘Jonagold’	‘Conference’
Porosity (%)	18.4 ± 4.4 (ab)	12.1 ± 3.0 (b)	25.4 ± 2.6 (a)	5.7 ± 1.4 (c)
Cell volume (mm^3^)	0.0045 ± 0.0004 (a)	0.0030 ± 0.0007 (b)	0.0052 ± 0.0004 (a)	0.0023 ± 0.0007 (b)
Cell surface/volume (mm^2^ mm^−3^)	16.27 ± 0.93 (ab)	22.0 ± 4.3 (a)	18.9 ± 1.4 (a)	12.1 ± 1.6 (b)
Equivalent spherical diameter (μm)	196.6 ± 7.0 (a)	172 ± 13 (b)	209.7 ± 3.5 (a)	159 ± 17 (b)
Cell count (mm^−3^)	181 ± 20 (b)	293 ± 82 (ab)	142 ± 8 (b)	411 ± 107 (a)
Cell elongation (-)	1.52 ± 0.07	1.72 ± 0.18	1.54 ± 0.02	1.52 ± 0.06
Cell-to-void area (relative to cell surface) (%)	37.0 ± 4.0 (a)	34.4 ± 3.7 (ab)	41.7 ± 4.3 (a)	21.8 ± 9.9 (b)
Void volume (mm^3^)	0.0019 ± 0.0008 (b)	0.0006 ± 0.0004 (c)	0.0047 ± 0.0003 (a)	0.00005 ± 0.00001 (c)
Void surface/volume (mm^2^ mm^−3^)	66 ± 14 (bc)	104 ± 28 (b)	50.3 ± 4.1 (c)	204 ± 18 (a)
Void equivalent spherical diameter (μm)	100 ± 11 (b)	71 ± 16 (c)	139 ± 12 (a)	39.4 ± 1.8 (d)
Weighted void count (mm^−3^)	77 ± 24 (b)	220 ± 120 (b)	28.0 ± 2.5 (b)	540 ± 190 (a)
Void elongation (-)	2.36 ± 0.46	3.23 ± 0.85	2.07 ± 0.21	2.17 ± 0.14
Void shape factor	2.032 ± 0.029 (ab)	2.12 ± 0.15 (ab)	2.29 ± 0.11 (a)	1.94 ± 0.15 (b)
Branching number	950 ± 330 (c)	2410 ± 760 (b)	570 ± 230 (c)	5500 ± 3200 (a)
Total void path length (mm mm^−3^)	28 ± 13 (b)	84 ± 35 (a)	13.1 ± 7.5 (b)	100 ± 32 (a)
Void anisotropy (-)	0.241 ± 0.097 (b)	0.39 ± 0.13 (ab)	0.468 ± 0.033 (a)	0.226 ± 0.072 (b)
Fragmentation index (mm^−1^)	22.3 ± 7.0 (b)	44.9 ± 13.9 (b)	6.5 ± 5.4 (c)	82.3 ± 8.5 (a)
Oxygen diffusion coefficient (10^−9^ m^2^ s^−1^)^a^	1.73 ± 0.5 (b)	2.73 ± 1.59 (b)	10.1 ± 5.2 (a)	0.28 ± 0.15 (c)

^a^Ho et al. (2010); Verboven et al. (2008)

The cell shape of the different apple and pear tissues are largely comparable. ‘Jonagold’ has the most uniform cell elongation, and ‘Kanzi’ has the most variable distribution of cell shapes. The cell-to-void area fraction is not significantly different between the apple cultivars ‘Braeburn’ (37.0 %), ‘Kanzi’ (34.4 %) and ‘Jonagold’ (41.7 %). This fraction is, however, much lower for ‘Conference’ pear (21.8 %), meaning that less than a quarter of the cell surfaces is exposed to intercellular spaces for gas exchange. This has important consequences with respect to metabolic gas exchange [3, 6].

### Characteristics of void networks in pome fruit cortex

**Fig. 3:**
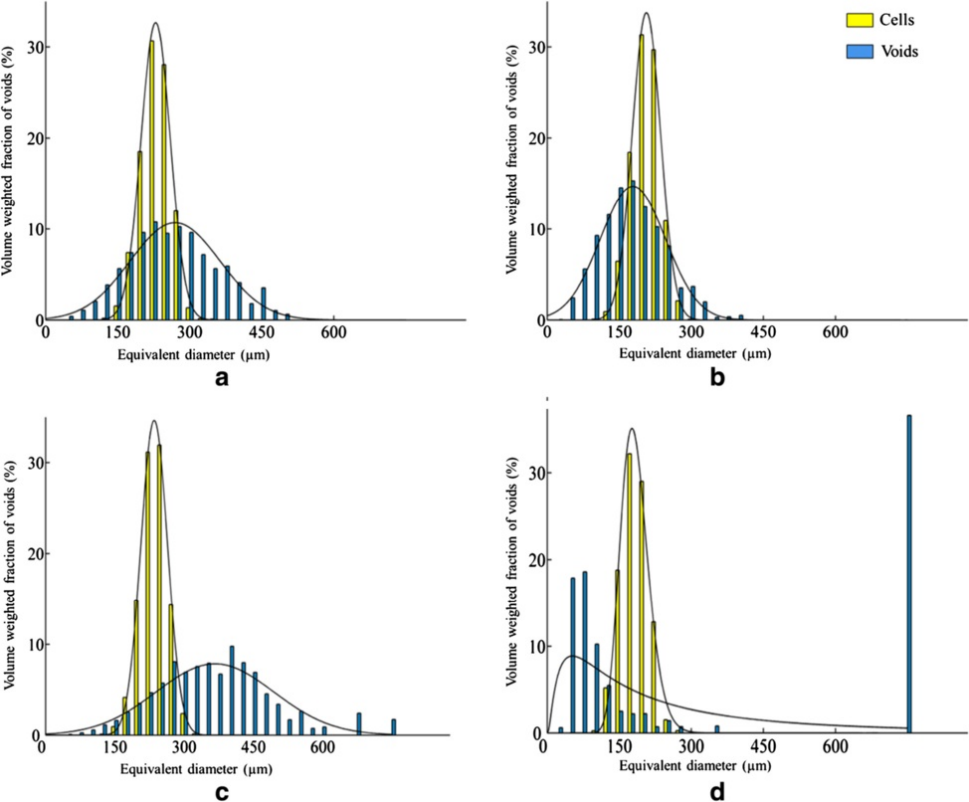
Cell (yellow) and void (blue) volume distributions for ‘Braeburn’ (**a**), ‘Kanzi’(**b**), ‘Jonagold’ (**c**) and ‘Conference’ (**d**), in which the relative distributions of equivalent diameters (μm) are volume weighted (%). Histograms are based on the image processing and analysis of 4 individual CT scans of cortex fruit tissue samples. The cells and voids were modelled by a normal distributions (*p >* 0.05), ‘Conference’ voids were modelled by a Weibull distribution

Application of the void isolation protocol resulted in 500 to 3000 voids in each 3D image for detailed quantification beyond previous more qualitative descriptions [6]. The size distributions of individual voids show a higher variation compared to those of the cells (Fig. 3). The equivalent spherical diameter of the voids ranges from 50 to over 500 μm. For the apple cultivars, ‘Kanzi’ manifests a relatively larger abundance of small voids, whereas the voids of ‘Jonagold’ are by far the largest, and are similar in volume as the cells of that cultivar (Table 1). The voids that were detected in ‘Conference’ pear samples are more uniformly distributed in terms of their size, and are substantially smaller than the voids in apple tissue. All fruit types have significantly different average void diameters.

We successfully fitted normal distributions to the void size histograms (*p*-value > 0.05) (Fig. 3). The void network of ‘Conference’ is clearly not normally distributed, due to a large number of small voids, as well as a small number of highly branched large voids, forming a large fraction of the void volume. We attempted to fit a Weibull distribution to the histogram, but due to the presence of one very large, interconnected void with an equivalent diameter of about 750 μm in one of the samples the distribution of the fit is not good.

Void volume shape characteristics of ‘Kanzi’ are more similar to those of pear, while ‘Braeburn’ has more irregular voids such as ‘Jonagold’. The statistical differences between these two groups of fruit are, however, less pronounced than for cell characteristics. The number of voids per mm^3^ (Table 1) is significantly higher for ‘Conference’ pear (540) compared to those of the apple samples (28 for ‘Jonagold’, 77 for ‘Braeburn’ and 220 voids ‘Kanzi’). The variability in void count is high in all fruit except ‘Jonagold’ that has the smallest number of voids. This characteristic is also a measure for connectivity of the network of air spaces in the tissue. A value 1 would mean that the air space is a continuous network throughout the tissue. The higher the number, the more disconnected the voids are. Clearly all fruit have a significantly disconnected void network, confirming earlier observations [6, 19].

The measured voids, being 2 to 3 times as long as they are wide, are more elongated (or narrower) than cells. The estimated void surface shape is significantly larger for ‘Jonagold’ than for ‘Conference’ pear but not different between apple genotypes. Differences are small but suggest that the surface area of individual voids in apple are on average more moulded to fit around cells, and ‘Conference’ voids are more tubular. The branching number of the ‘Conference’ void system (5500 per mm^3^) is significantly higher than that of the apple genotypes (<2410 per mm^3^). Although the global porosity value is lower for pear tissue, it implies that the void network in ‘Conference’ is intricately more complex and consists of more connected pathways than for any of the examined apple cultivars.

**Fig. 4:**
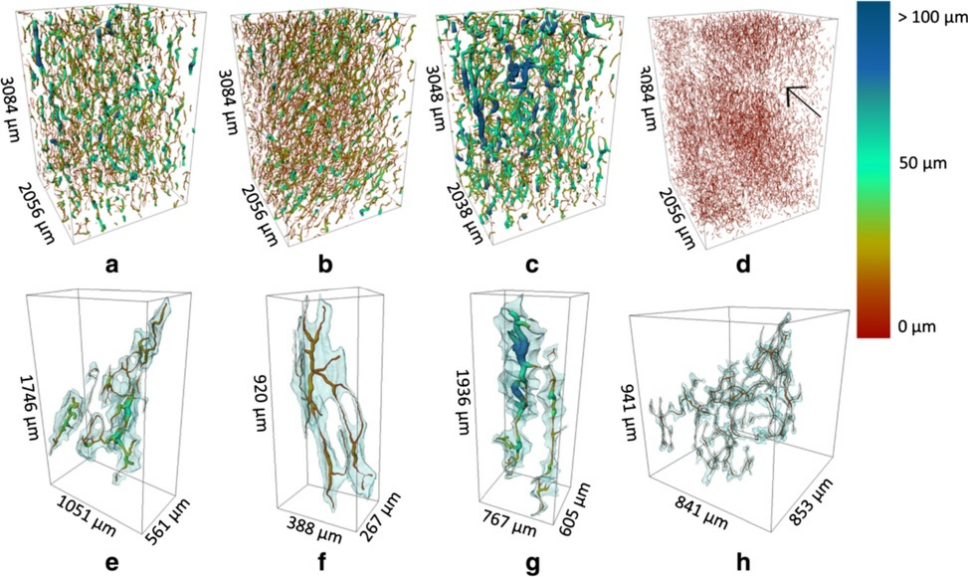
Void network models for ‘Braeburn’ (**a**), ‘Kanzi’ (**b**), ‘Jonagold’ (**c**) and ‘Conference’ (**d**) showing void topology and void network branching as well as local thicknesses of the voids expressed by the color scale. The arrow in the ‘Conference’ image indicates the presence of a stone cells, with a local absence of surrounding voids. Details of the void network models for a single void, show the original void volume (transparent blue) and the calculated void network. For these models, large void volumes were chosen to illustrate the void connectivity. The dimensions of the box illustrate the spatial extent of the void network. Plots **e** to **h** present a single connected void in each of the corresponding void networks in plots **a** to **d**, demo,strating variations in size and connectivity

The void network plots show the essential void architecture and connectivity of the different studied genotypes (Fig. 4). It is clear that ‘Jonagold’ has the widest voids, with the largest local thicknesses compared to ‘Braeburn’ and ‘Kanzi’. For ‘Conference’ pear, a void network is shown in which the typical configuration with absence of voids around the stone cells can be recognized. More detailed images of the void networks (Fig. 4e-h) reveal the distinct microarchitecture of the voids, with a large spread in thickness and connectivity.

Based on the extracted skeletons, we can calculate a theoretical total path length in which metabolic gases can be transported throughout the void networks of the different genotypes. In a cubic millimetre, we estimate that there is 100 mm of path length allocated for gas diffusion in ‘Conference’ pear, through the large labyrinth of airspaces in between the cells (Table 1). The total void length is considerably larger for ‘Conference’ and ‘Kanzi’ than for ‘Braeburn’ and ‘Jonagold’. Finally, the longer void network in ‘Conference’ is accompanied by a high degree of fragmentation. The fragmentation is less in ‘Braeburn’ and ‘Kanzi’, and lowest in ‘Jonagold’.

### Relation to aeration properties of fruit parenchyma tissue

The most important physiological function of the intercellular space in fruit is to facilitate metabolic gas transport [3, 5, 6]. We will now investigate whether the microstructural properties can be related to the different genotypes in terms of gas transport. Apparent oxygen diffusivity is a measure for the characteristic rate of exchange of this respiratory gas in cortex tissue of fruit. It has been shown that this parameter significantly affects respiratory metabolism in fruit during postharvest storage [4]. Oxygen diffusion coefficients for cortex tissue of ‘Braeburn’, ‘Kanzi’, ‘Jonagold’, and ‘Conference’, obtained from previous work on the same cultivars [5] are listed in Table 1. ‘Conference’ has significantly smaller diffusivity values than the apple cultivars. For apple, ‘Jonagold’ has the largest diffusivity which is significantly larger than that of the other genotypes.

**Fig. 5:**
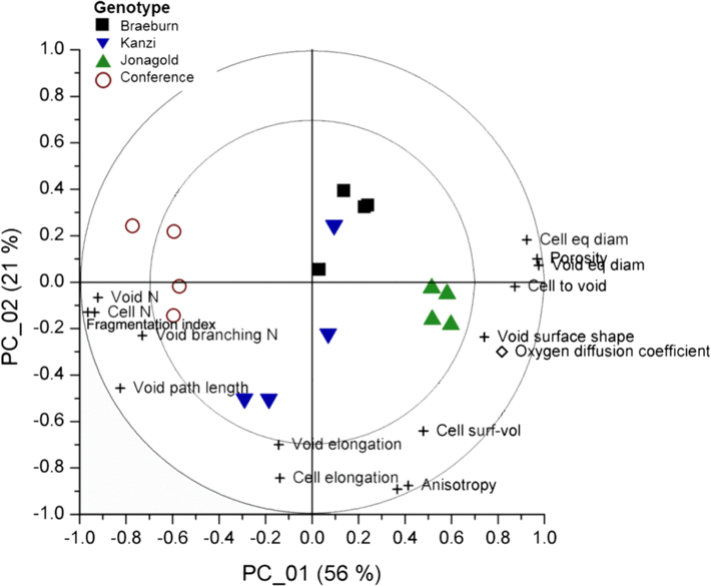
PCA biplot of the samples of 4 genotypes ( ‘Braeburn’,  ‘Kanzi’,  ‘Jonagold’,  ‘Conference’), showing the location and grouping of the samples in terms of their microstructural characteristics, the measured variables which should be interpreted as vectors with their origin in (0,0). Correlation loadings () situated between the circles (70 and 100 % explained variance limits) are considered most important for explaining the variability with respect to the principal components shown. Correlation loadings based on literature data () for effective oxygen diffusion are added to the biplot. Variables with loadings situated in proximity of each other are correlated. Loadings that make a 90° angle are said to be mostly uncorrelated. Loadings with an 180° angle are inversely related. In this case, an increased porosity is associated with a lower number of cells and voids, with increased respective volumes. Although ‘Conference’ samples have low porosity, they are characterized by a high number of voids, with a high degree of branching. Microstructural shape determinants such as elongation of cells and voids and anisotropy of the tissue are related to each other, but mostly unrelated to other microstructural descriptors. The first 2 PC’s explained 77 % of the total X-variance

The relationship between the measured microstructural parameters and effective diffusivity of the tissue is represented graphically in a biplot after principal component analysis (PCA, Fig. 5). In this PCA biplot, the scores represent the apple samples; the correlation loadings the microstructural features and functional property (O_2_ diffusivity). The latter should be interpreted as vectors starting in the origin and ending in the corresponding symbols. Correlation loading vectors that point in the same or opposite direction indicate a large positive or negative correlation; correlation loading vectors that are perpendicular to each other are not correlated at all. If a correlation loading vector points in the direction of a score (apple sample) then this implies that the latter is characterized by a positive value of the corresponding microstructural feature or biophysical property. Correlation loading vectors that end within both concentric circles can be considered as relevant.

The first and second principal component accounted for 56 % and 21 % of the total variability, respectively, which indicates some redundancy in presumably the microstructural features. An increased porosity is associated with a smaller number of cells and voids and with large equivalent diameters. Void path length scales with branching number and fragmentation of the voids. Although ‘Conference’ samples have low porosity, they are characterized by a high number of voids (also indicative of a strongly fragmented void network), with a high degree of branching, but low cell-to-void surface area. At the other end of the spectrum, ‘Jonagold’ is positioned, while the other apple cultivars have an intermediate position between the two extremes.

Microstructural shape determinants such as elongation of cells and voids and anisotropy of the tissue are correlated to each other, but mostly uncorrelated to other microstructural descriptors and functional properties. Void connectivity, cell connectivity and cell surface to volume ratio do not appear to play significant roles in determining aeration properties of fruit cortex tissue. The effective oxygen diffusivity is positively correlated to porosity (and its related parameters) and the surface area and shape of the voids, and negatively correlates to the number of voids and cells, as well as the number and length of branches in the void network and the degree of fragmentation of the network. The value of effective oxygen diffusivity is several orders of magnitude smaller than that of air (${D}_{O_2,a}$ = 2.15 × 10^−5^ m^2^ s^−1^). Also between genotypes differences cover different orders of magnitude: ‘Jonagold’ has the highest average value, followed by ‘Kanzi’ and ‘Braebrun’; the lowest value is for ‘Conference’. Considerable variability can also be noticed for each genotype.

### Correlation formula of effective diffusivity of fruit parenchyma tissue

Effective property models exist in the form of parametric equations for two-component materials that account for structural effects for a range of standard structures [27, 28]. Of these, the Effective Medium Theory (EMT) and Maxwell-type structure models are more like the structure observed in parenchyma tissue. Also these models rely only on porosity and diffusivity in air and cells to compute the effective diffusivity. Cellular diffusivity is approximated by the diffusivity of water (${D}_{O_2,w}$) multiplied by solubility: ${D}_{O_2,w}\cdot R\cdot T\cdot {H}_{O_2}$ = 9.3 × 10^−11^ m^2^ s^−1^, with ${D}_{O_2,w}$ = 2.75 × 10^−9^ m^2^ s^−1^, *R* (J mol^−1^ K^−1^) the universal gas constant, *T* temperature (K) and ${H}_{O_2}$ (0.0137 mol m^3^ kPa^−1^) the Henry constant for water. The resulting values of effective diffusivity (using the Maxwell-Eucken model, Eq. 5 below) are 1.1 × 10^−10^ m^2^ s^−1^ for ‘Conference’, 1.3 × 10^−10^ m^2^ s^−1^ for ‘Kanzi’, 1.6 × 10^−10^ m^2^ s^−1^ for ‘Braeburn’ and 1.9 × 10^−10^ m^2^ s^−1^ for ‘Jonagold’. The resulting values of effective diffusivity approach that of oxygen through the cells and are much smaller than those measured. Thus, these approximations over emphasize the cellular diffusion pathway.

New models for effective diffusivity of parenchyma tissue are required and should be a weighted sum of the parallel porous media model [29] and a heterogeneous conductivity model [28] according to the method of [30]:

1 $$ {D}_{O_2, eff}=\frac{1}{\frac{w}{D_{O_2, eff}^{par}}+\frac{\left(1-w\right)}{D_{O_2, eff}^{ser}}} $$

2 $$ {D}_{O_2, eff}^{par}=\varepsilon {D}_{O_2,a}/{\tau}^2 $$

3 $$ {D}_{O_2, eff}^{ser}=\frac{\left(1-\varepsilon \right){D}_{O_2,w}\cdot R\cdot T\cdot {H}_{O_2}+\varepsilon {D}_{O_2,a}\frac{3{D}_{O_2,w}}{2{D}_{O_2,w}\cdot R\cdot T\cdot {H}_{O_2}+{D}_{O_2,a}}}{D_{O_2,w}\cdot R\cdot T\cdot {H}_{O_2}+{D}_{O_2,a}\frac{3{D}_{O_2,w}\cdot R\cdot T\cdot {H}_{O_2}}{2{D}_{O_2,w}\cdot R\cdot T\cdot {H}_{O_2}+{D}_{O_2,a}}} $$

with *ε* the porosity, *τ* the tortuosity and *w* the weighing factor. ${D}_{O_2,a}$ and ${D}_{O_2,w}$ are the oxygen diffusivity in air and water, and $\cdot R\cdot T\cdot {H}_{O_2}$ is the factor accounting for solubility of oxygen in water. A Maxwell-Eucken formulation [28] is used for the serial contribution in Eq. 3, assuming a dispersed volume of voids in a continuous matrix of cells. Using the average experimental values of effective diffusivity of each genotype, we can roughly estimate the weighing factor *w*. The value of *w* for all apple genotypes is above 0.90, and for ‘Conference’ 0.65. Even with relatively high contributions of porous medium diffusion through connected pores, a contribution as low as a few percentages for the series diffusion across cells between disconnected pores can bring the effective diffusivity down with almost 2 orders of magnitude. With 35 % in pear, the effective diffusivity drops 3 orders of magnitude. The resulting effective value is also very sensitive to the weighing factor, and helps explaining why a high variability of effective diffusivity is observed depending on local degree of connectivity of the void network in tissues.

## Discussion

### Tissue structure characterisation by automated processing of X-ray images is possible

Plant structure analysis using automated image analysis aims to link genotypes to phenotypes and study plant growth and physiology at several spatial and temporal resolutions. Recent software solutions from cell to canopy level studies have been organized in an online database [31] (www.plant-image-analysis.org). Most common applications are leaf analyses and shoot or root meristems, basically flat or superficial structures, which can be imaged by conventional 2D light, confocal or electron microscopy, and for which 2D area measurements are sufficient to characterize the essential cellular anatomy. However, for imaging and analyzing parenchyma cells of bulky plant organs, it is more challenging to achieve a spatial resolution that is sufficient to resolve characteristic features at certain depths [32]. Current works have only achieved manual segmentation [33] or using approximate image analysis methods [34–36]. Dhondt et al. (2010) analysed micro CT images of the *Arabidopsis* hypocotyl quantitatively; however, fixation of the sample was needed prior to X-ray CT imaging, after which individual cell volumes could be measured. Our method measures actual cell volumes of in vivo samples, as well as that of the intact void space automatically.

The workflow we developed to analyse the 3-D microstructure of plant tissue has been applied successfully to isolate individual cells in their natural state without the need for extensive sample preparation. The methodology is also applicable to sample sizes of an order of magnitude of 1 mm^3^ with currently available desktop X-ray microtomography techniques [22], thus exceeding that of previous attempts with at least 1 order of magnitude [18].

The smallest voids (i.e., isolated air space volumes) that we observed measured 6.9 × 10^−6^ mm^3^, corresponding to 51 image voxels. Smaller isolated voids were by default removed by the noise filter in processing the images.

Even though the sample size was large compared to a typical cell size, it was recognized that relatively large voids may exist in fruit tissue. The used protocol disfavours such void sizes in the range of sample size, as these have a higher probability of intersecting the volume boundaries. Larger sample sizes are possible; however, this comes at the expense of reduced image resolution and loss of image quality [19, 22]. Progress in X-ray CT technology will be required to overcome this limitation. In this study the size already exceeded by more than 10 fold that of the representative elementary volume (REV = 1.3 mm^3^) determined for apple tissue [19]. However, this REV was determined for global porosity only, not for evaluating other individual microstructural features, and, therefore, should be revisited. In the case of ‘Jonagold’, excluding the boundary-intersecting voids led to 64 % of the void volume being discarded in the analysis, compared to 34, 42 and 49 % for ‘Braeburn’, ‘Kanzi’ and ‘Conference’. A corrective measure based on assigning a larger weight to larger voids in the counting procedure was, therefore, implemented.

The watershed separation of cells favours the segmentation of regularly shaped spherical cells. The volume fraction of cellular tissue that was removed at different image processing steps is shown in Table 2. A large fraction of the tissue volume was removed for touching image borders (40 %). A smaller volume of cell material (roughly 20 to 30 %) remained unseparated and was removed for further analysis. The removal of these clustered cells may have affected the results, by excluding the more extreme cell shapes. However, considering the large amount of cells that was obtained from each tissue sample, the results are likely to be representative for the fruit tissue. This was not the case for the void space analysis as the void volume and shape descriptors were measured on the original X-ray CT images, without watershed analysis.

**Table 2:** Remaining volume fraction of cellular tissue at two specific steps in the + image processing protocol for cell isolation. The volume fraction is expressed in terms of the total cellular volume in the unprocessed images. The remaining volume fraction after removing clusters was used for analyzing the individual cells

	‘Braeburn’	‘Kanzi’	‘Jonagold’	‘Conference’
Volume fraction after removing broken cells and cells touching borders (%)	60.28 ± 1.83	60.00 ± 1.41	54.41 ± 5.03	58.67 ± 1.83
Volume fraction after removing cell clusters (%)	39.79 ± 8.70	26.75 ± 18.17	33.60 ± 8.13	28.91 ± 11.77

### Fragmentation of the void network reduces effective tissue diffusivity up to 3 orders of magnitude

Although the effective diffusivity is highly correlated to porosity (Fig. 5), other tissue-specific characteristics need to be considered. Tortuosity is the most obvious choice. Using available theoretical porous media models [8], the tissue tortuosity ranges between 1.4 and 4.2 for the porosity range of 25.4 to 5.7 %. As a result, the computed effective diffusivity value will be on average 2.7 × 10^−6^ m^2^ s^−1^ for ‘Jonagold’ and 7.0 × 10^−8^ m^2^ s^−1^ for ‘Conference’, or 200 to 300 times smaller than the average experimental values. Cortex tissue of fruit, and most likely any plant tissue, thus cannot be considered as a conventional porous medium for gas exchange where porosity is the main parameter. The measured void shape factor could also be interpreted as tortuosity (Table 1). The square of this value could be a fair estimate of the tortuosity factor [9]. This leads to high effective diffusivity values for all the genotypes, namely 1.0 × 10^−6^ m^2^ s^−1^, 9.9 × 10^−7^ m^2^ s^−1^, 5.9 × 10^−7^ m^2^ s^−1^ and 3.4 × 10^−7^ m^2^ s^−1^ for ‘Jonagold’, ‘Braeburn’, ‘Kanzi’ and ‘Conference’, respectively. These values are between 100 and 1000 times larger than the experimental values. It must be noted that the void shape factor has a positive correlation with oxygen diffusivity in Fig. 5; which does not comply with this porous medium theory. It can thus be possible that this shape factor is not a true indicator of tortuosity or, more likely, that other factors dominate the diffusion process.

The average path length per void ranges from 0.19 mm for ‘Conference’ to 0.43 mm for ‘Jonagold’. Thus, in cortex tissues with a thickness of several cm it is very unlikely to find a fully connected aeration network through the voids justifying porous medium diffusion; therefore the porous medium assumption breaks down and disconnectivity of the network must be taken into account. The number of voids per mm^3^ volume indeed expresses the fact that the void network is disconnected. The number ranges from 28 per mm^3^ for ‘Jonagold’ to 540 per mm^3^ for ‘Conference’. Figure 7 clearly shows the significance of this parameter for oxygen diffusivity, along with the fragmentation index of pores.

Because the void network is disconnected, diffusion through the cells must be taken into account for effective diffusion in the tissue. The effective diffusivity would simply become zero otherwise as gasses cannot diffuse through the disconnected void network. This explains why the fractional area of cells exposed to the airspaces are positively correlated to effective diffusivity (Fig. 5). The larger this property in the tissue, the better gas diffuses across the cell-void interface, which is an important pathway in low porosity tissue with disconnected pores. The value of specific void surface area is indeed high in all genotypes (Table 1). The equivalent diameters of cell and voids are for the same reason significant parameters. In tissue with disconnected voids, the mechanism of gas diffusion no longer follows a simple parallel mechanism that states that the effective diffusivity is the porosity-weighted sum of the diffusion in the air spaces and the cells, but the relative thickness of voids and cells in a serial layer model are also important. Previously proposed parallel model for effective diffusivity of tissue [10, 29] should therefore be improved to include effects of serial diffusion through the cellular or cell wall pathway. Such model equation is hypothesized in Eq. (3).

Equation (3) is a new parametric equation for tissue oxygen diffusivity that is presented based on a combination of similar equations already available in the literature and complies with the current observations of measured diffusivity. As such, it does not present a new modeling approach. In the past we have developed and applied a new modelling approach that directly computes the tissue effective diffusivity from the 3D microstructural geometry of the porous structure [3, 37, 38]. While this is a validated approach, it relies on the availability of 3D micro-CT images that need to be processed for computational use. Furthermore, it is enlightening to quantify which microstructural properties precisely are determinant for the diffusivity. That is now made most clear by, first, the PCA analysis, and second, the proposed parametric equation that in principle could be solved without the need for actual microscopic images, but does require the relevant parameters to be determined by imaging or other means.”

It is difficult to find a good correlation between the weighing factor in Eq. (3) and the average structural parameters in Table 1. It is clear that it is strongly depended on the fragmentation properties of the void network. Rather than developing statistical correlations, significant progress has been made with physical models that compute effective diffusivity directly from tissue structure that matches to experimentally determined values [3]. This modeling approach relies directly on 3D computer models of the exact tissue anatomy without a need for tissue structure analysis. Such modeling can, however, be further supported using the analysis tool developed here: the statistical properties can be used as a basis to generate virtual tissues for such computational models. Different plant tissue generation algorithms are currently being developed for such purpose [39–42]. Also, in other plant aeration studies, the modeling approach has been applied successfully [43–45].

### Relation of tissue properties to hypoxic response of the different fruit genotypes

Optimal storage conditions of the genotypes studied here differ considerably. For long-term storage, ‘Jonagold’ are conventionally kept at 1 kPa O_2_, Kanzi at 2 kPa O_2_ and ‘Conference’ and ‘Braeburn’ at 2.5 kPa O_2_ [46]. Indeed, the effect of storage O_2_ partial pressures on the risk of fermentation inside the fruit can be calculated using the values of values oxygen diffusivity in combination with the respective respiration kinetics of the genotype [4, 47]. Such analysis shows that ‘Jonagold’ (with relatively higher diffusivity) can be stored at low O_2_ partial pressure, while high O_2_ partial pressures are required for ‘Kanzi’, ‘Braeburn’ and ‘Conference’ (with lower tissue diffusivity).

## Conclusions

The methodology described in this article yields distributions of cell size and shape as well as a quantitative description of tissue architecture and the void network that can lead to better plant anatomy understanding and models. By counting and characterising single cells in in vivo fruit samples and the geometrical information thus obtained, we realized a detailed insight in fruit microstructure in relation to tissue aeration. We found considerable differences of the structural configuration of 3 different apple cultivars and the pear cultivar that affected oxygen diffusivity significantly. Such microstructural information is valuable for explaining and possibly even predicting gas-exchange related disorders. We propose to use this method as a research tool to create detailed tissue libraries, containing different 3D geometric models (‘cybertypes’), that could be used for generating in silico tissue models to support plant research in general. On a practical note, the protocols presented here were developed in a commercial software for 3D image processing and will require reprogramming when implemented in other environments.

## Methods

### Apple and pear fruit samples

‘Conference’ pears (*Pyrus communis*), ‘Jonagold’, ‘Kanzi’ and ‘Braeburn’ apples (*Malus* × *domestica* Borkh.) were grown at the experimental orchard of the Research Station of Fruit Growing in Velm (Belgium) and harvested on 17/09/2010, 25/09/2006, 04/10/2010 and 27/10/2010, respectively, which was in the optimal commercial picking window for long term storage for each cultivar, determined by Flanders Centre of Postharvest Technology (VCBT, Leuven, Belgium).

The time in between harvest and the actual X-ray CT scans ranged from 19 days for ‘Braeburn’ and 2 months for ‘Conference’ and ‘Jonagold’. During the storage period, fruits was kept in cool rooms at the optimal long term storage temperature, under normal atmosphere (1 °C for ‘Braeburn’ and 4 °C for ‘Kanzi’) or controlled atmosphere conditions (1 kPa O2, 2 kPa CO2 and 0.8 °C for ‘Jonagold’; – 2.5 kPa O2, < 0.8 kPa CO2, -1 °C for ‘Conference’). Sampling of the apples was performed in a standardized manner. A cylindrical sample with a diameter of 5.98 mm was excised radially along the fruit equator using a cork bore. In the case of the apples, this was taken from the fruit’s sun exposed side, while for pear it was taken at a random position. A subsample (5 mm height) was cut with a scalpel by removing the tissue directly under the skin and within the core (pericarp), sampling only the so-called cortex (hypanthium or accessory tissue of apple fruit). Samples from 4 different fruits were measured for each cultivar.

### X-ray CT scans

X-ray CT images of fresh ‘Conference’, ‘Braeburn’ and ‘Kanzi’ fruit tissue were obtained using a SkyScan 1172 system (Bruker microCT, Kontich, Belgium) as reported by Herremans et al. (2013b). The 3D microstructure of ‘Jonagold’ cortex tissue was obtained from synchrotron radiation tomography images recorded at beamline ID19 of the European Synchrotron Radiation Facility (ESRF, Grenoble, France) as described by Verboven et al. (2008). Reconstructing the datasets resulted in a 3D stack consisting of isotropic voxels with a single pixel measuring 5.14 μm for the images of ‘Braeburn’, ‘Kanzi’ and ‘Conference’ and 5.08 μm for ‘Jonagold’ images. This pixel resolution was found sufficient for visualizing air spaces and cell outlines in fruit parenchyma tissue. The 3D air space volume and shape was the same in images taken at 0.7 μm and 5 μm pixel size [6]; while lower resolution (pixel size > 5 μm) results in loss of image quality [19]. The pixel values correspond to the linear X-ray attenuation coefficient, displayed as a grey scale value calibrated between 0 and 255. Although there are certainly differences in terms of resolution and signal-to-noise ratio when comparing desktop X-ray CT to synchrotron X-ray CT images, due to the similar pixel sizes and thanks to the customized image processing protocol the resulting 3D image stacks gave equivalent results.

### Tissue anatomy analysis algorithm

#### Image preprocessing

The micro-CT image datasets (10.28 x 10.28 x 5.39 mm^3^) were trimmed on the lateral sides in order to remove the surrounding polystyrene foam regions from the images. Hereby also 6 to 7 cell layers were excluded to remove damaged cells at the edge as a result of cutting the samples. The remaining prismatic volume measured 2.1 x 2.1 x 3.1 mm^3^, a volume which was found representative of cortex tissue [19]. The cells in the fruit cortex tissue could be readily segmented from the intercellular airspace by means of Otsu thresholding [48] because of the excellent contrast between the two distinct regions in the images.

#### Cell isolation

**Fig. 6:**
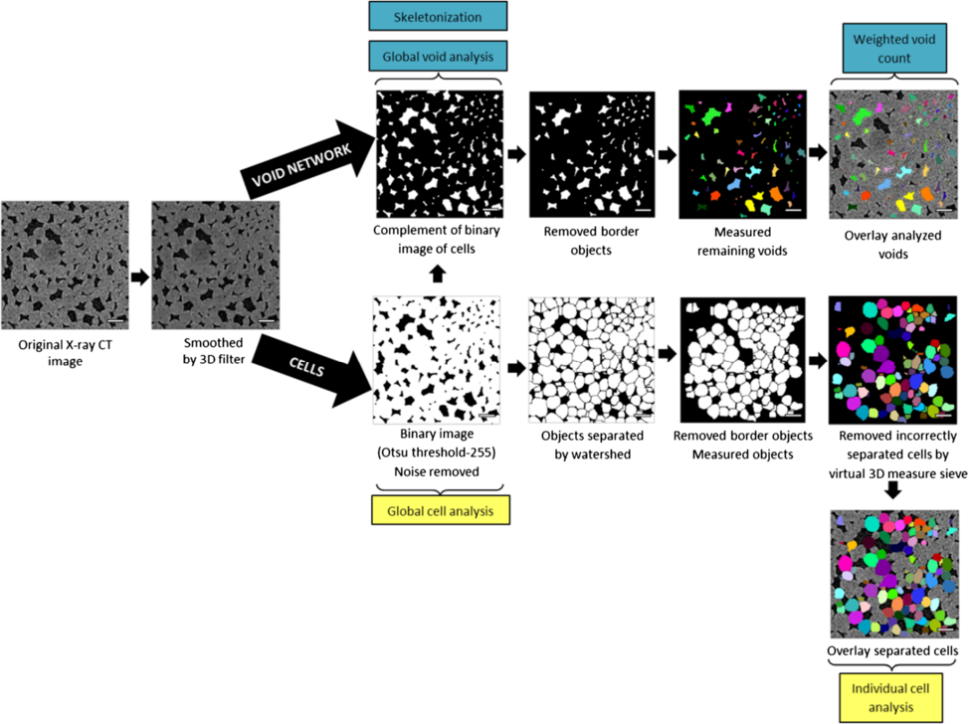
Cross-sections of ‘Braeburn’ microstructure illustrating consecutive steps of the cell isolation and void characterization protocol. The images obtained by means of X-ray CT are cropped and filtered before segmentation into the cellular matrix and void network. These complementary binary images are processed in a separate workflow. Voids intersecting the border of the image are removed and resulting voids are characterized in 3D. The cellular matrix is separated in individual objects by the watershed separation algorithm. Objects touching the image border are removed and remaining objects are measured. Objects of which the length and sphericity do not fall within the user-defined range of cellular characteristic are removed. The analyzed void network and separated cells are overlayed with the original image. Scale bars in images indicate 250 μm

The 3D region in the image that was identified as cells, needs to be subdivided into individual cells. The different steps of the algorithm are explained in Fig. 6. To differentiate neighbouring cells in the binary image, we optimized a specialized image processing tool called watershed separation, particularly suited for separating touching, convex features [49]. The algorithm works by calculating the Euclidean distance map (EDM) of the binary image, in which grayscales represent the distance from that pixel to the nearest black (background) pixel, regardless of direction. The EDM can be seen as a height chart, producing a mountain peak at the center of each of the cells. Upon flooding of the terrain, water will shed down the peaks and run downhill until it reaches the bottom of the valley. Locations in the local valleys are reached by water running down from different peaks. Removing those points separates the peaks, and correspondingly separates the various cells in the binary images [50, 51].

After the watershed separation, all cells that intersected the dataset borders were removed in order to exclude incomplete individual cells from the later morphological analysis. Based on literature data [6, 52] and manual cell segmentation, the average cell size and the cell sphericity index (ratio of the surface area of a sphere, with the same volume as the cell, to the surface area of the cell) were used to construct a virtual sieve to discard incorrectly segmented cell particles or unseparated clumps of cells. All individual objects with a length larger than 400 μm (for different apple cultivars, the average representative diameter was 200 μm [52]), and with a sphericity index lower than 0.75 were removed from the 3D model, keeping only correctly separated single cells. The protocol was validated by manually segmenting individual cells in 3 datasets (Additional file 1: S2). The calculated volume distributions were compared statistically. No significant differences (significance level 0.05) were found for the automatic isolation protocol and the manual expert segmentation. The cell separation algorithm was implemented in Avizo Fire (VSG, France) and is detailed in Additional file 1: S1.

#### Void network analysis

**Fig. 7:**
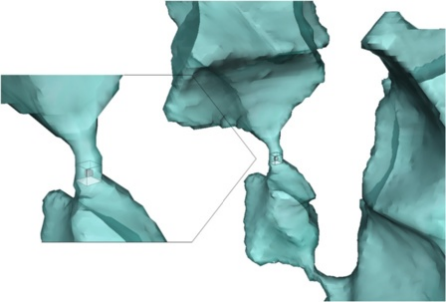
Volume rendering of a part of the void space in Jonagold apple cortex tissue, showing voids (*blue*) with bulky volumes that are connected through narrow passages (detail). Neighbouring void voxels that have at least 1 face, 1 edge or 1 corner in common (26 pixel connectivity – shown in detail), are considered to be connected and thus belong to the same void

The air space can be considered as the ensemble of individual voids that are interconnected by narrower passages (Fig. 7). To identify the individual voids, it suffices to evaluate 3D connectivity of each void voxel with a structuring element of the surrounding 26 voxels. This means voxels with at least 1 common vertex are considered to be connected to each other, and belong to the same void.

In order to correctly count the number of individual voids, an adjustment has to be made to correct for the voids intersecting the image boundary. As these voids can be connected to each other outside the edges of the field of view, the void number will be overestimated. A solution consists of performing a weighted count of all objects that do not touch any edge [49]. Such a strategy is known as the "Miles-Lantuéjoul correction” [53]. The weighted count compensates for the likelihood that a void with certain dimensions would touch the edge of the dataset. The weight-adjusted count of each void is a real number, larger than one defined by:

4 $$ Coun{t}_{adj}=\frac{I_x{I}_y{I}_z}{\left({I}_x-{P}_x\right)\left({I}_y-{P}_y\right)\left({I}_z-{P}_z\right)} $$

where *I*_*x*_*, I*_*y*_ and *I*_*z*_ are the dimensions of the image in the *x, y* and *z* directions, and *P*_*x*_*, P*_*y*_ and *P*_*z*_ are the maximum projected dimensions of the voids in those directions, which were derived by calculating the respective Feret diameters of each void, as if the voids were measured by means of a calliper, to determine the distance between planes parallel to the 3D image stack, and tangential to the void. Small voids that are not likely to touch or cross the edge of the image thus count for approximately one each; while large voids that are more likely to touch or cross the edge of the image count for more than one.

Additionally to the void analysis, the construction of the air space network skeleton reveals how voids are connected in the tissue. The dedicated skeletonization algorithm [54] was implemented, based on a thinning procedure which removes voxel by voxel from the binarized void space until only a string of connected voxels remains. The primary output is a spatial graph representation of the centrelines, maintaining the essential void topology and the Euclidean distance to the nearest void-to-cell boundary is stored at every point in the spatial graph object as a local thickness attribute. The 3D binary image stacks of isolated voids were obtained and further processed using the skeletonization module in Avizo Fire software (VSG, France) and is detailed in Additional file 1: S1.

#### Calculation of structural parameters

Particular structural parameters are relevant quantitative descriptors of the air space and cells of the different tissues. We calculated porosity, the volume, surface and equivalent diameter distributions of cells and voids, the volumetric count and elongation of cells and voids directly from the individual cell and void images. The fraction of the surface of cells that was exposed to the void space was also calculated.

To express the shape of the void surface in terms of its protrusions between the cells, we calculated the void surface shape as the ratio of the actually measured void surface area *A*_*v*_ and the surface area of a cylinder (*A*_*v,cyl*_)with the same volume *V* and equivalent diameter *d*_*eq*_:

5 $$ \tau =\frac{A_v}{A_{v,cyl}}=\frac{A_v{d}_{eq}}{4V} $$

The total path length of the branches in the entire void phase was estimated by adding the lengths of all branches. The number of branching points is extracted from the topology of the void skeleton to evaluate its complexity.

Anisotropy indicates the existence of a preferential orientation of the void spaces in the cortex tissue. It is calculated by performing a mean intercept length analysis, in which a grid of lines is basically sent through the binary void space over a large number of 3D angles. The number and length of intercepts between the lines of the grid and the void/cell interphase is evaluated at all these angles. Differing mean intercept lengths for different 3D angles indicate an anisotropic spatial organisation [55].

The fragmentation index is calculated by the proportion of changes in surface area to changes in volume of the void space by image dilation [56]. A relatively lower value signifies a better connected void network. The numerical value, however, does not have a physical meaning.

The structural parameters were calculated in commercial softwares CTAn 1.12.0.0 (Bruker microCT, Kontich, Belgium) and Avizo Fire edition 8.0.0 (VSG, Bordeaux, France).

#### Oxygen diffusivity of fruit parenchyma tissue

The parenchyma tissue diffusivity of apple and pear fruit samples was measured using the measurement setup developed by Ho et al. (2006a). The values of effective tissue diffusivity of the inner cortex for the different apple genotypes were previously measured by [37]; those of pear by [57].

Statistical analysis was performed using the SAS Enterprise guide software 4.3 (SAS Institute, Cary, NC, USA) and Unscrambler software version 10.1 (CAMO Software, Oslo, Norway).

## Availability of supporting data

S1. Tissue anatomy analysis procedures in Avizo Fire software

S2. Validation of the automatic cell isolation algorithm

The data sets supporting the results of this article will be made available at http://www.biw.kuleuven.be/biosyst/mebios/mebiosdownloads.

## Additional file

Additional file 1:
**S1.** Tissue anatomy analysis procedures in Avizo Fire software. **S2.** Validation of the automatic cell isolation algorithm. (DOCX 1180 kb)

## References

[1] Armstrong W (1979). Aeration in higher plants. Adv Bot Res.

[2] Franck C, Lammertyn J, Ho QT, Verboven P, Verlinden B, Nicolaï BM (2007). Browning disorders in pear fruit. Postharvest Biol Technol.

[3] Ho QT, Verboven P, Verlinden BE, Herremans E, Wevers M, Carmeliet J, Nicolaï BM (2011). A three-dimensional multiscale model for gas exchange in fruit. Plant Physiol.

[4] Ho QT, Verboven P, Verlinden BE, Schenk A, Nicolaï BM (2013). Controlled atmosphere storage may lead to local ATP deficiency in apple. Postharvest Biol Technol.

[5] Ho QT, Verboven P, Verlinden BE, Schenk A, Delele MA, Rolletschek H, Vercammen J, Nicolaï BM (2010). Genotype effects on internal gas gradients in apple fruit. J Exp Bot.

[6] Verboven P, Kerckhofs G, Mebatsion HK, Ho QT, Temst K, Wevers M, Cloetens P, Nicolaï BM (2008). Three-dimensional gas exchange pathways in pome fruit characterized by synchrotron x-ray computed tomography. Plant Physiol.

[7] Boudreau BP (1996). The diffusive tortuosity of fine-grained unlithified sediments. Geochim Cosmochim Acta.

[8] Shen L, Chen Z (2007). Critical review of the impact of tortuosity on diffusion. Chem Eng Sci.

[9] Gommes CJ, Blacher S, Dunsmuir JH, Tsou AH: Practical Methods for Measuring the Tortuosity of Porous Materials from Binary or Gray-Tone Tomographic Reconstructions. AIChE J. 2009;55:2000–2012

[10] Pham QT, Schotsmans W, Ho QT, Verlinden BE, Verboven P, Nicolaï BM (2008). Simultaneous measurement of neon diffusivity and skin resistance of “Braeburn” and “Jonica” apples. Postharvest Biol Technol.

[11] Harada T, Kurahashi W, Yanai M, Wakasa Y, Satoh T (2005). Involvement of cell proliferation and cell enlargement in increasing the fruit size of Malus species. Sci Hortic (Amsterdam).

[12] Malladi A, Hirst PM (2010). Increase in fruit size of a spontaneous mutant of “Gala” apple (Malus x domestica Borkh.) is facilitated by altered cell production and enhanced cell size. J Exp Bot.

[13] Chalermchat Y, Malangone L, Dejmek P (2010). Electropermeabilization of apple tissue: Effect of cell size, cell size distribution and cell orientation. Biosyst Eng.

[14] Wood DF, De J, Berrios J, Venet C (2006). Microstructure of the processing of black beans (Phaseolus vulgaris L.) from purée to drum-dried flakes. Scanning.

[15] Jarvis MC, Briggs SPH, Knox JP. Intercellular adhesion and cell separation in plants. Plant Cell Environ. 2003;44:977–989

[16] Varela P, Salvador A, Fiszman S (2007). Changes in apple tissue with storage time: Rheological, textural and microstructural analyses. J Food Eng.

[17] Laurienzo P, Cammarota G, Di Stasio M, Gentile G, Laurino C, Volpe MG (2013). Microstructure and olfactory quality of apples de-hydrated by innovative technologies. J Food Eng.

[18] Wuyts N, Palauqui J, Conejero G, Verdeil J, Granier C, Massonnet C (2010). High-contrast three-dimensional imaging of the Arabidopsis leaf enables the analysis of cell dimensions in the epidermis and mesophyll. Plant Methods.

[19] Mendoza F, Verboven P, Mebatsion HK, Kerckhofs G, Wevers M, Nicolaï B (2007). Three-dimensional pore space quantification of apple tissue using X-ray computed microtomography. Planta.

[20] Dhondt S, Wuyts N, Inzé D (2013). Cell to whole-plant phenotyping: the best is yet to come. Trends Plant Sci.

[21] Ting VJL, Silcock P, Bremer PJ, Biasioli F (2013). X-ray micro-computer tomographic method to visualize the microstructure of different apple cultivars. J Food Sci.

[22] Herremans E, Verboven P, Bongaers E, Estrade P, Verlinden BE, Wevers M (2013). Characterisation of “Braeburn” browning disorder by means of X-ray micro-CT. Postharvest Biol Technol.

[23] Verboven P, Herremans E, Helfen L, Ho QT, Abera M, Baumbach T, Wevers M, Nicolaï BM (2014). Synchrotron X-ray computed laminography of the three-dimensional anatomy of tomato leaves. Plant J.

[24] Dhondt S, Vanhaeren H, Van Loo D, Cnudde V, Inzé D (2010). Plant structure visualization by high-resolution X-ray computed tomography. Trends Plant Sci.

[25] Gonzalez RC, Woods RE (2008). Digital Image Processing*.* Third edit.

[26] Pridmore TP, French AP, Pound MP (2012). What lies beneath: underlying assumptions in bioimage analysis. Trends Plant Sci.

[27] Wang M, Pan N (2008). Predictions of effective physical properties of complex multiphase materials. Mater Sci Eng R Reports.

[28] Wang J, Carson JK, North MF, Cleland DJ (2008). A new structural model of effective thermal conductivity for heterogeneous materials with co-continuous phases. Int J Heat Mass Transf.

[29] Ho QT, Verboven P, Verlinden BE, Lammertyn J, Vandewalle S, Nicolaï BM (2008). A continuum model for metabolic gas exchange in pear fruit. PLoS Comput Biol.

[30] Krischer O (1963). Die Wissenschaftlichen Grundlagen Der Trocknungstechnik.

[31] Lobet G, Draye X, Périlleux C (2013). An online database for plant image analysis software tools. Plant Methods.

[32] Zehbe R, Haibel A, Riesemeier H, Gross U, Kirkpatrick CJ, Schubert H, Brochhausen C (2010). Going beyond histology. Synchrotron micro-computed tomography as a methodology for biological tissue characterization: from tissue morphology to individual cells. J R Soc Interface.

[33] Gray J, Kolesik P, Høj P, Coombe B (1999). Confocal measurement of the three-dimensional size and shape of plant parenchyma cells in a developing fruit tissue. Plant J.

[34] Cheniclet C, Rong WY, Causse M, Frangne N, Bolling L, Bordeaux VS, Ornon V (2005). Cell Expansion and Endoreduplication Show a Large Genetic Variability in Pericarp and Contribute Strongly to Tomato Fruit Growth 1. Plant Physiol.

[35] Legland D, Devaux M-F, Bouchet B, Guillon F, Lahaye M (2012). Cartography of cell morphology in tomato pericarp at the fruit scale. J Microsc.

[36] Devaux M-F, Bouchet B, Legland D, Guillon F, Lahaye M (2008). Macro-vision and grey level granulometry for quantification of tomato pericarp structure. Postharvest Biol Technol.

[37] Ho QT, Verboven P, Verlinden BE, Nicolaï BM (2010). A model for gas transport in pear fruit at multiple scales. J Exp Bot.

[38] Ho QT, Verboven P, Mebatsion HK, Verlinden BE, Vandewalle S, Nicolaï BM (2009). Microscale mechanisms of gas exchange in fruit tissue. New Phytol.

[39] Dupuy L, Mackenzie J, Haseloff J (2010). Coordination of plant cell division and expansion in a simple morphogenetic system. Proc Natl Acad Sci U S A.

[40] Merks RMH, Guravage M, Inzé D, Beemster GTS (2011). VirtualLeaf: an open-source framework for cell-based modeling of plant tissue growth and development. Plant Physiol.

[41] Prusinkiewicz P, Runions A (2012). Computational models of plant development and form. New Phytol.

[42] Abera MK, Verboven P, Herremans E, Defraeye T, Fanta SW, Ho QT, Carmeliet J, Nicolai BM (2014). 3D Virtual Pome Fruit Tissue Generation Based on Cell Growth Modeling. Food Bioprocess Technol.

[43] Armstrong W, Strange M, Cringle S, Beckett P (1994). Microelectrode and Modelling Study of Oxygen Distribution in Roots. Ann Bot.

[44] Verboven P, Herremans E, Borisjuk L, Helfen L, Ho QT, Tschiersch H, Fuchs J, Nicolaï BM, Rolletschek H (2013). Void space inside the developing seed of Brassica napus and the modelling of its function. New Phytol.

[45] Verboven P, Pedersen O, Herremans E, Ho QT, Nicolai B, Colmer TD, Teakle N (2012). Root aeration via aerenchymatous phellem: three-dimensional micro-imaging and radial O2 profiles in Melilotus siculus. New Phytol.

[46] Schenk A: Voorbereidingen voor de pluk. In: Bart Nicolai, Ann Schenk, editors. Year report 2013. Leuven, Belgium: Flanders Centre of Postharvest Technology; p. 1–19

[47] Ho QT, Rogge S, Verboven P, Verlinden BE, Nicolaï BM: Stochastic modelling for virtual engineering of controlled atmosphere storage of fruit. J Food Eng 2015. doi:10.1016/j.jfoodeng.2015.07.003.

[48] Otsu N (1979). A Threshold Selection Method from Gray-Level Histograms. IEEE Trans Symstems Man, Cybern.

[49] Russ JC (2005). Image Analysis of Food Microstructure.

[50] Herremans E, Bongaers E, Estrade P, Gondek E, Hertog M, Jakubczyk E, Nguyen Do Trong N, Rizzolo A, Saeys W, Spinelli L, Torricelli A, Vanoli M, Verboven P, Nicolaï B (2013). Microstructure–texture relationships of aerated sugar gels: Novel measurement techniques for analysis and control. Innov Food Sci Emerg Technol.

[51] Soille P (2002). Discrete Geometry for Computer Imagery.

[52] Schotsmans W, Verlinden BE, Lammertyn J, Nicolaï BM (2004). The relationship between gas transport properties and the histology of apple. J Sci Food Agric.

[53] Miles R: On the elimination of edge effects in planar sampling. In: Harding EF, Kendall DG, editors. Stochastic Geometry. London, UK: John Wiley & Sons; 1974:228–247.

[54] Fouard C, Malandain G, Prohaska S, Westerhoff M (2006). Blockwise processing applied to brain microvascular network study. IEEE Trans Med Imaging.

[55] Odgaard A (1997). Three-dimensional methods for quantification of cancellous bone architecture. Bone.

[56] Hahn M, Vogel M, Pompesius-Kempa M, Delling G (1992). Trabecular bone pattern factor--a new parameter for simple quantification of bone microarchitecture. Bone.

[57] Ho QT, Verlinden BE, Verboven P, Nicolaï BM (2006). Gas diffusion properties at different positions in the pear. Postharvest Biol Technol.

