# Supplementary material for: Automatic analysis of the 3-D microstructure of fruit parenchyma tissue using X-ray micro-CT explains differences in aeration
Source: BMC Plant Biol. 2015 Oct 30;15:264. doi: 10.1186/s12870-015-0650-y (PMC4628266; doi:10.1186/s12870-015-0650-y)
Supplement: Additional file 1: — S1. Tissue anatomy analysis procedures in Avizo Fire software. S2. Validation of the automatic cell isolation algorithm. (DOCX 1180 kb) [file 12870_2015_650_MOESM1_ESM.docx]

# Automatic analysis of the 3-D microstructure of fruit parenchyma tissue using X-ray micro-CT explains differences in aeration

Els Herremans^1^, Pieter Verboven^1^, Bert E. Verlinden^2^, Dennis Cantre^1^, Metadel Abera^1^, Martine Wevers^3^, Bart M. Nicolaï^1,2^

^1^BIOSYST-MeBioS, KU Leuven, Willem de Croylaan 42, 3001 Leuven, Belgium

^2^Flanders Centre of Postharvest Technology, Willem de Croylaan 42, 3001 Leuven, Belgium

^3^MTM, KU Leuven, Kasteelpark Arenberg 44, 3001 Leuven, Belgium

Corresponding author:

Pieter Verboven

BIOSYST-MeBioS, KU Leuven, Willem de Croylaan 42, 3001 Leuven, Belgium

Tel.: +32 16 32 14 53

E-mail: pieter.verboven@biw.kuleuven.be

# Supplementary material

# S1. Tissue anatomy analysis procedures in Avizo Fire software

An overview of the software image processing protocol is given in Figure S1. The X-ray CT images were filtered by an edge-preserving smoothing filter (Figure S2). This filter smooths out the difference between gray levels of neighboring voxels. However, it does not smear out the edges because the smoothing operation is reduced or stopped in the vicinity of edges. The filter models the physical process of diffusion (Weickert et al, 1998). The stop time determines how long the diffusion runs. The longer it runs, the smoother the image becomes. The time step determines how accurately this process is sampled. The contrast parameter determines how much the diffusion process depends on the image gradient, i.e., how much the smoothing is stopped near edges. A value of 0 makes the diffusion independent of the image gradient and smooths out the edges, a large value prevents smoothing in all edge-like regions. In order to make the diffusion process more stable, the image is prefiltered by a Gaussian filter.

The filtered images were binarised by applying the Otsu threshold in the “Segmentation editor” (Figure S3), after which noise was removed (“Remove islands”), i.e., black or white objects smaller than 27 voxels (Figure S4).

To differentiate neighbouring cells in the binary images, we applied the “binseparate” algorithm. This module created a binary image containing the regional maxima of the input distance map image, which were merged within the contrast variation given as parameter (Figure S5). These regional maxima were kept as markers for the watershed algorithm that isolated individual cells.

After the watershed separation, all cells that intersected the dataset borders were removed by applying the “Borderkill” operation (Figure S6). Next, the remaining cells were characterised in 3D by applying the “I_analyze” module (Figure S7) in which a large number of 3D parameters were calculated for each individual cell. The output of this module is a database (Figure S8) that was used to filter out unwanted objects based on any of the measured parameters. In this case, objects that did not meet size (length < 400 µm) and shape (sphericity > 1) criteria were removed, as these cells were most likely not separated correctly.

The void analysis (Figure S1, workflow on the right) involved the same modules. The binarised images, were now analysed for the void phase. Voids intersecting the borders were removed (“Border_kill”), and the remaining objects were measured (“I_analyze”).


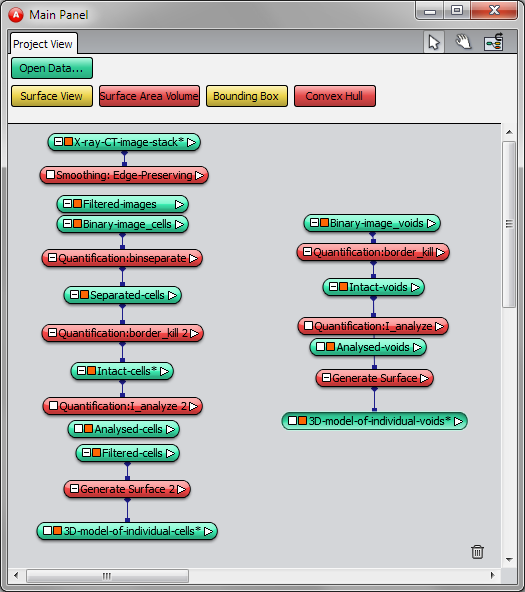


Figure S1. Workflow of the tissue analysis algorithm in Avizo software. The green icons are image datasets, the red icons are computations that are performed on the images. The workflow on the left starts with the reconstructed X-ray CT images of apple microstructure, and results in the 3D model of automatically separated cells. The workflow on the right uses the binarised images to characterise the individual voids, and generate 3D model of these.


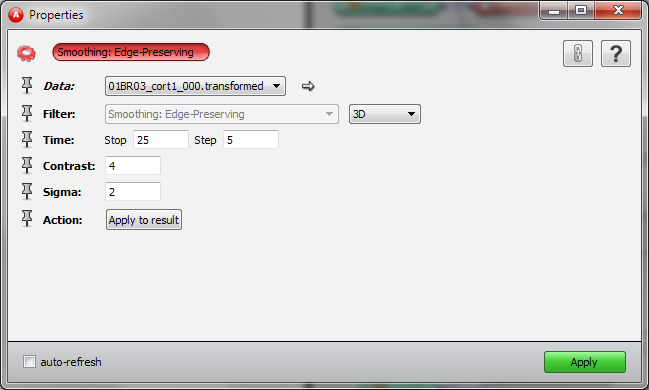


Figure S2. Screenshot of the “Edge-preserving filter”.


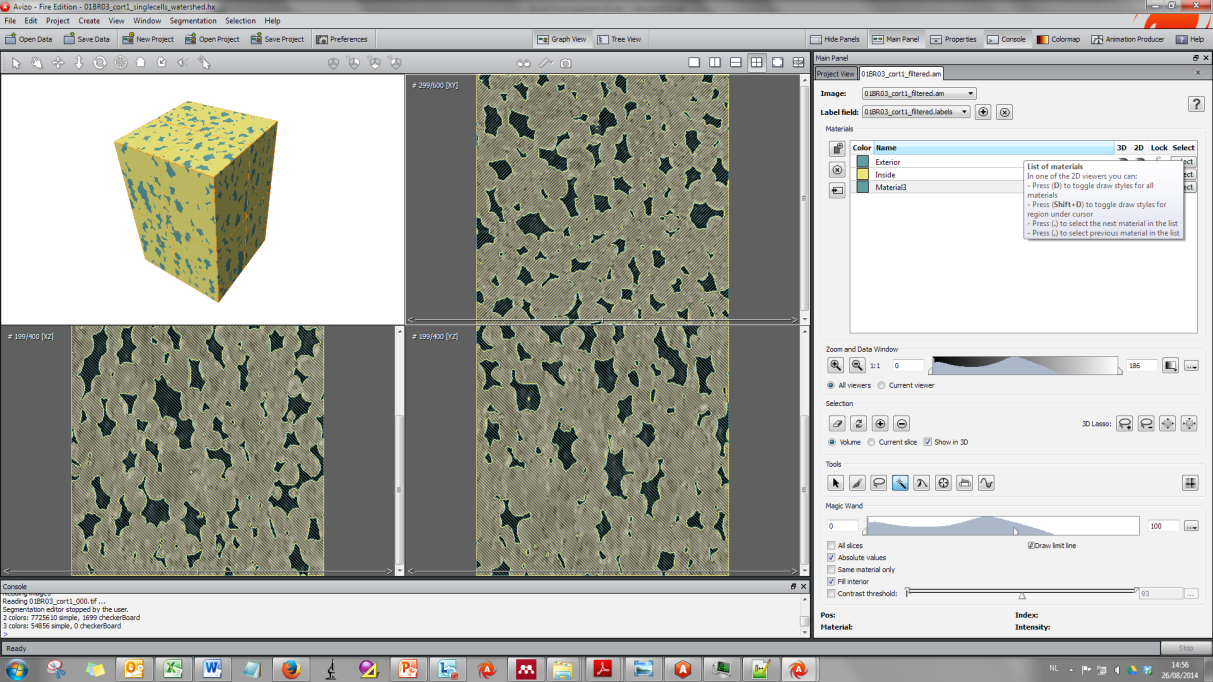


Figure S3. Screenshot of the binarised dataset in the “Segmentation editor”.


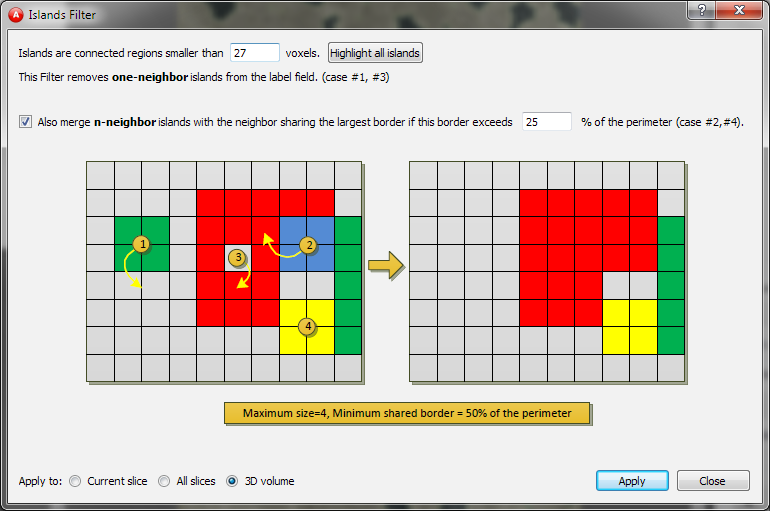


Figure S4. Screenshot of noise removal by the “Islands filter”.


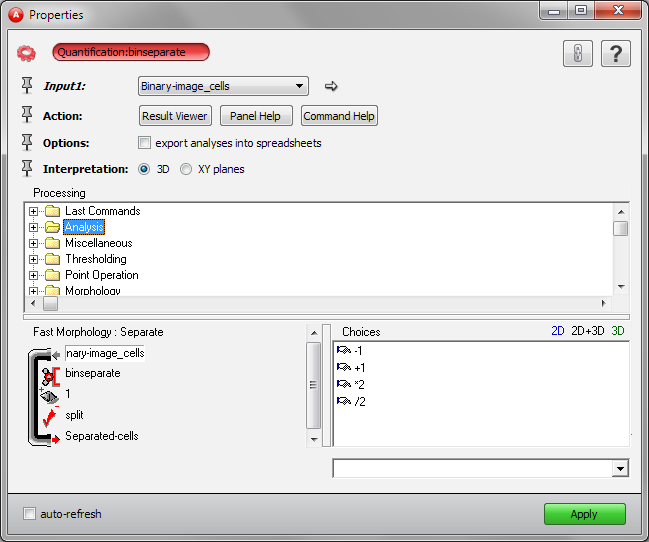


(a)

(b)

Figure S5. (a) Screenshot of the “Binseparate” module and (b) the principle of merged maxima shown on an image profile sample (VSG, 2013).


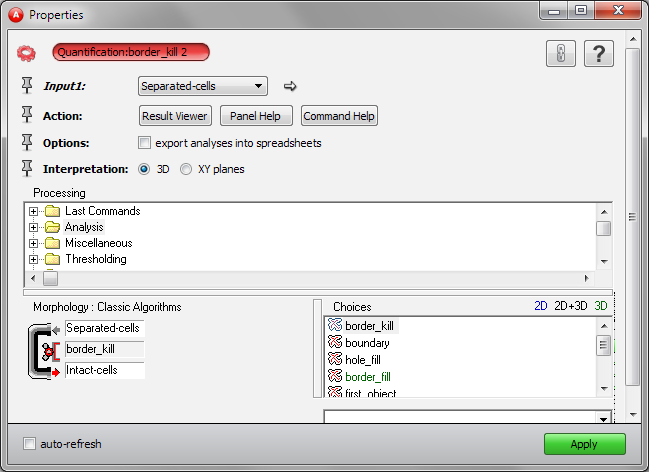


Figure S6. Screenshot of the “Border_kill” module that was used to remove objects that were intersected by the image datastack boundary.


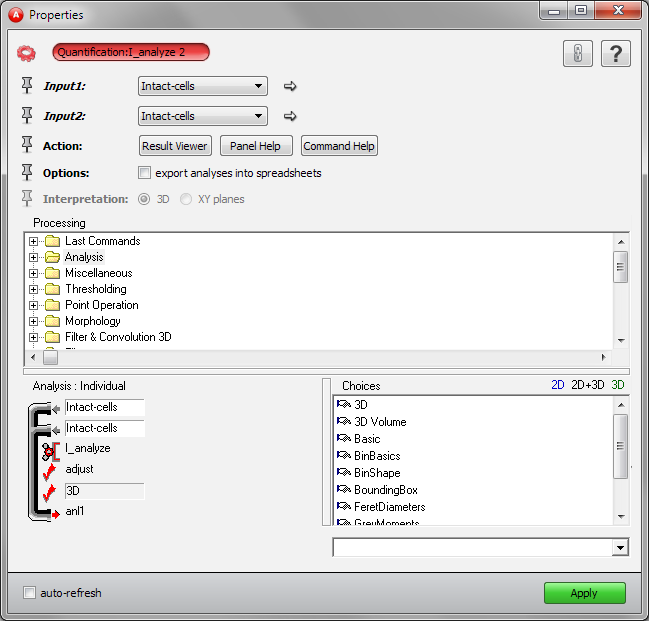


Figure S7: Screenshot of the “I_analyze” module in which 3D measurements were performed.


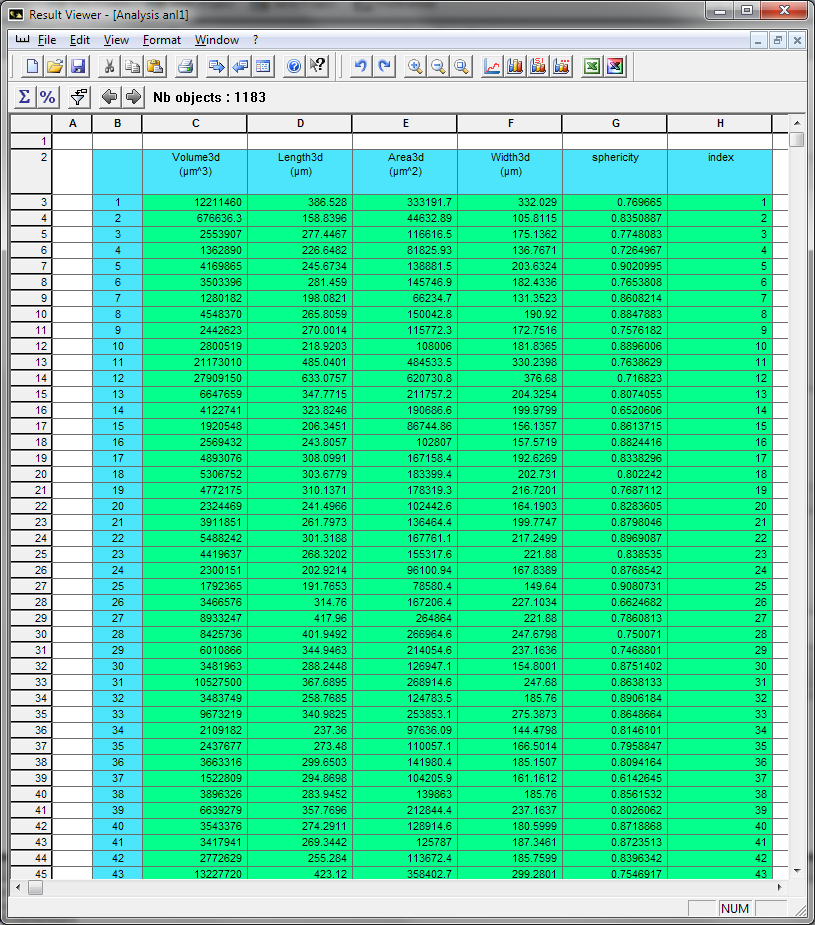


(a)


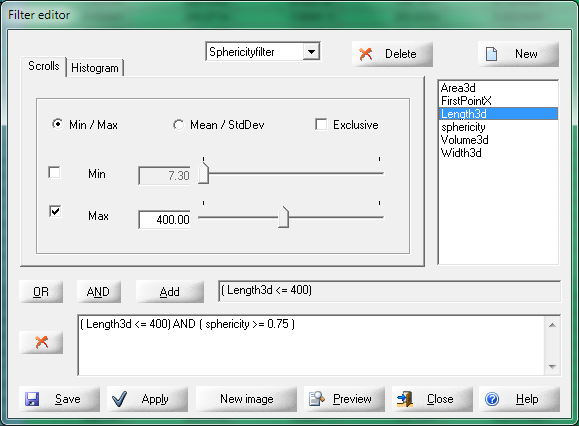


(b)

Figure S8. (a) Screenshot of the 3D measurements that were obtained for each individual cell.(b) Screenshot of the “Filter editor” that was used to remove objects from the database and the 3D image stack.

# S2. Validation of the automatic cell isolation algorithm

The cell separation and analysis protocol was validated by manually segmenting individual intact cells in 3 datasets of Kanzi apple fruit. The averaged volume distributions using the automatic and manual results are plotted in Figure S9 for different parameters used in the automatic protocol:

- The watershed (WS) algorithm can be used with different contrast factors (WS0, WS1 and WS2, Figure S5b) that define the threshold level for defining a peak in the height chart. It takes care of noise or uncertainly in the height data. Increasing WS numbers relaxes the criterion for counting a peak and smoothes the algorithm resulting in a smaller number of isolated cells that are larger in size. WS1 is preferred over WS0. WS2 does not improve isolation.
- Second, we apply smoothing of the isolated volumes to obtain more ellipsoidal cell shapes. Smoothing has an optimum on 3 pixel basis; increased values of the smoothing factor reduce the accuracy of the method. .

The calculated volume distributions using WS1 and 3 smoothing pixels were compared statistically to manually segmented datasets. No significant differences (significance level 0.05) were found for the automatic isolation protocol and the manual expert segmentation.

Figure S9. Cumulative number distribution of cells as a function of the equivalent diameter of the intact cells for different isolation parameters (WS: watershed contrast factor (0,1,3), Smooth: smoothing of isolated volumes: 0, 3 or 6 pixels).
